# Four-years change of BMI and waist circumference are associated with metabolic syndrome in middle-aged and elderly Chinese

**DOI:** 10.1038/s41598-024-60172-w

**Published:** 2024-05-03

**Authors:** Xiaoyun Zhang, Ying Wang, Yuqing Li, Jiaofeng Gui, Yujin Mei, Xue Yang, Haiyang Liu, Lei-lei Guo, Jinlong Li, Yunxiao Lei, Xiaoping Li, Lu Sun, Liu Yang, Ting Yuan, Congzhi Wang, Dongmei Zhang, Jing Li, Mingming Liu, Ying Hua, Lin Zhang

**Affiliations:** 1 Department of Graduate School, Wannan Medical College, 22 Wenchang West Road, Higher Education Park, Wuhu City, An Hui Province People’s Republic of China; 2 Student Health Center, Wannan Medical College, 22 Wenchang West Road, Higher Education Park, Wuhu City, An Hui Province People’s Republic of China; 3 Department of Surgical Nursing, School of Nursing, Jinzhou Medical University, No.40, Section 3, Songpo Road, Linghe District, Jinzhou City, Liaoning Province People’s Republic of China; 4 Department of Occupational and Environmental Health, Key Laboratory of Occupational Health and Safety for Coal Industry in Hebei Province, School of Public Health, North China University of Science and Technology, Tangshan, Hebei Province People’s Republic of China; 5 Obstetrics and Gynecology Nursing, School of Nursing, Wannan Medical College, 22 Wenchang West Road, Higher Education Park, Wuhu City, An Hui Province People’s Republic of China; 6 Department of Emergency and Critical Care Nursing, School of Nursing, Wannan Medical College, 22 Wenchang West Road, Higher Education Park, Wuhu City, An Hui Province People’s Republic of China; 7 Department of Internal Medicine Nursing, School of Nursing, Wannan Medical College, 22 Wenchang West Road, Higher Education Park, Wuhu City, An Hui Province People’s Republic of China; 8 Department of Pediatric Nursing, School of Nursing, Wannan Medical College, 22 Wenchang West Road, Higher Education Park, Wuhu City, An Hui Province People’s Republic of China; 9 Department of Surgical Nursing, School of Nursing, Wannan Medical College, 22 Wenchang West Road, Higher Education Park, Wuhu City, An Hui Province People’s Republic of China; 10 Rehabilitation Nursing, School of Nursing, Wannan Medical College, 22 Wenchang West Road, Higher Education Park, Wuhu City, An Hui Province People’s Republic of China

**Keywords:** Metabolic syndrome, Middle-aged and elderly, Body mass index, Waist circumference, National cohort study, Endocrinology, Risk factors, Predictive markers

## Abstract

The purpose of the study was to determine whether changes in body mass index (BMI) and waist circumference (WC) in middle-aged and elderly Chinese are associated with metabolic syndrome. In this cohort investigation, 3697 middle-aged and elderly people aged 45 or over were recruited from the China Health and Retirement Longitudinal Study (CHARLS). The National Cholesterol Education Program Adult Treatment Panel III (2005) defined metabolic syndrome (MetS). With Cox regression analysis, we calculated hazard ratio (HR) and 95% confidence intervals (CIs) for MetS based on BMI-WC change categories. To assess the prevalence of MetS, the changes in BMI and WC levels were classified into four quartiles based on their relative and absolute changes. In subjects whose BMI and WC decreased (HR = 0.338; 95% CIs 0.264, 0.433) as well as those whose BMI increased and their WC decreased (HR = 0.375; 95% CIs 0.228, 0.499), metabolic syndrome risk was significantly lower compared with those with increases in both BMI and WC. Regarding the absolute changes in BMI, the lowest percentile of BMI was significantly lower in both males (HR = 0.302; 95% CIs 0.204, 0.448) and females (HR = 0.486; 95% CIs 0.354, 0.667) for the risk of metabolic syndrome. Similar results were observed in the absolute changes in WC, with the lowest quantile of WC having a significant impact on MetS risk in males (HR = 0.170; 95% CIs 0.107, 0.270) and females (HR = 0.303; 95% CIs 0.217, 0.424). The risk of metabolic syndrome was significantly associated with changes in BMI and WC in middle-aged and elderly Chinese. A reduced BMI and WC are associated with lower metabolic syndrome risks in middle-aged and elderly people.

## Introduction

Metabolic syndrome (MetS) is a collection of metabolic disorders characterized by insulin resistance, primarily manifested as obesity (especially abdominal obesity), hyperglycemia, hypertension, and dyslipidemia^1,2^. Currently, it is one of the most prevalent noncommunicable diseases. A number of chronic illnesses, including cancer^3,4^, nonalcoholic fatty liver disease^5^, type 2 diabetes^6^, and cardiovascular disease^7^ have been linked to MetS. According to different diagnostic criteria, the global prevalence of MetS varies from 12.5% to 31.4%^8^. The standardized incidence rate of metabolic syndrome is 31.1% in the data from China Nutrition and Health Surveillance (2015–2017), which includes 130,018 residents who are 20 years of age or older^9^. The incidence rates and prevalence rates of MetS are increasing worldwide due to the development of the social economy and the change of lifestyle, as well as the rapid development of population aging, especially in countries where obesity and the so-called Western diet (unhealthy) are prevalent^10–12^.

As one of the most common manifestations of metabolic syndrome and a marker of adipose tissue dysfunction, abdominal obesity is crucial for clinical diagnosis^13^. Due to the excessive deposition of abdominal fat and the limited regulatory function of subcutaneous adipose tissue energy storage, excessive chemical energy flows to the liver and skeletal muscles, leading to an increased risk of metabolic disorders^14,15^. It is well-known that direct evaluation of obesity and fat distribution through computer tomography (CT) or magnetic resonance imaging (MRI) is the gold standard, but this method is both expensive and complex for the general population^16^. In recent years, increasing epidemiological evidence has shown that simple and feasible anthropometric methods such as body mass index (BMI), waist circumference (WC), waist-to-height ratio (WHtR) and relative fat mass (RFM) can be used to predict the likelihood of MetS^17–19^. Among these indicators, BMI is the preferred indicator for evaluating general obesity, but BMI alone is an insufficient biomarker for abdominal obesity as it cannot distinguish between different body components (muscle and fat accumulation)^20^. The International Diabetes Foundation, National Institutes of Health, and WHO recommend the WC as a screening tool for metabolic and cardiovascular risks^21,22^. As a simple method for evaluating abdominal obesity, it can further refine the adverse health risks characterized by BMI and is easy to standardize and apply clinically^23^. NCEP-ATP III proposes to use abdominal obesity as the main parameter for determining MetS and recommends measuring WC instead of BMI^24^. It is well-known that weight reduction can reduce the risk of MetS and improve metabolic profiles^25^. However, weight is composed of body fat and muscle mass, and for any given BMI, the variation in WC is significant. Compared to those with lower waist circumferences, adults with higher waist circumferences are at higher risk of poor health^26^.

So far, the relationship between BMI, WC, and MetS risk has been confirmed in some cross-sectional and cohort studies. However, there are relatively few studies on the impact of BMI and WC changes on the MetS incidence rate. Therefore, our objective is to investigate the influence of alterations in BMI and WC on the occurrence rate of MetS among middle-aged and elderly individuals between 2011 and 2015, particularly in patients with varying BMI and WC changes, via a longitudinal study utilizing the data from the China Longitudinal Study of Health and Retirement (CHARLS). Additionally, this research carried out a stratified analysis according to sex, taking into account the disparities in MetS prevalence between sex and optimal BMI and WC cutoff points.

## Materials and methods

### Study design and setting

The data utilized in this study is sourced from the China Health and Retirement Longitudinal Study (CHARLS), a nationwide cohort study that specifically targets the middle-aged and elderly demographics in China^27^. With a cohort of 17,596 individuals ranging in age from 45 to 101, the CHARLS started in 2011 (Waves 1) and collected data in 2013 (Waves 2) and 2015 (Waves 3). Every two years, participants will complete structured questionnaires and face-to-face computer-assisted personal interviews (CAPIs). In the absence of any direct engagement with individuals, all information is openly available as microdata on the website http://charls.pku.edu.cn/index/zh-cn.html. The Ethics Committee of Peking University's China Center for Economic Research granted approval to the study after all participants had given their informed consent prior to data collection.

### Participants

**Figure 1:**
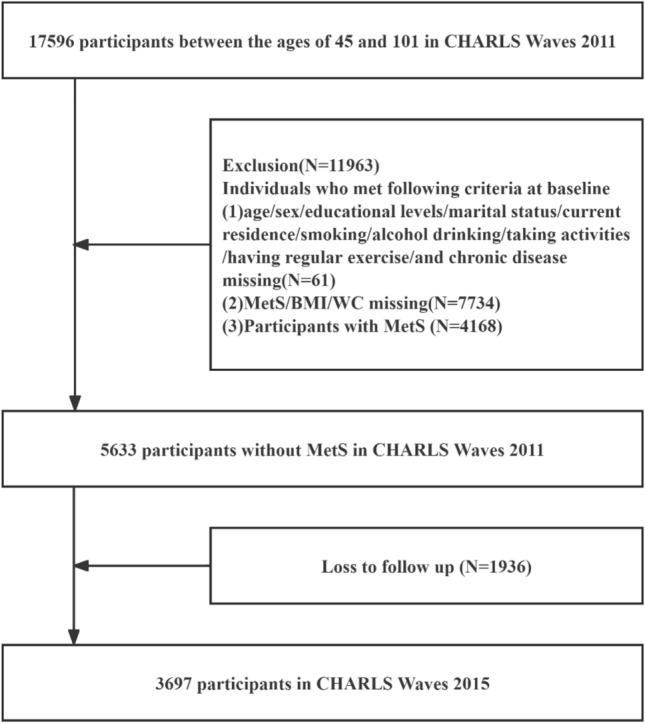
Flowchart of the study participants.

This study used data from the China Longitudinal Study on Health and Retirement (CHARLS) and selected participants from Waves 1 and Waves 3. After missing data subjects had been excluded, a total of 3697 individuals completed the baseline and follow-up surveys from 2011 to 2015. The average age of the 3697 individuals that participated in CHARLS was 58.42 years (standard deviation SD = 9.04). Males had a mean age of 60.17 years (SD = 8.80) while females had a mean age of 56.38 years (SD = 8.88). Figure 1 shows a flowchart of the study participants.

### Definition of metabolic syndrome

The definition and diagnostic criteria of MetS were proposed by NCEP ATP III (2005)^28,29^. According to the standard Chinese definition^30^, components of MetS are divided into five categories: WC ≥ 80 cm for women and ≥ 90 cm for men; TG levels of ≥ 150 mg/dl; HDL-C levels of < 40 mg/dl for men and < 50 mg/dl for women; SBP of ≥ 130 mmHg and/or DBP of ≥ 85 mmHg or using antihypertensive therapy; FPG levels of ≥ 100 mg/dl or using antidiabetic medications or self-reported medical history of diabetes. When three of the five listed characteristics are present, a diagnosis of MetS can be made.

### Measurement of BMI and WC

Body mass index (BMI) was used to measure participants’ weight; BMI = weight (kg) / [height (m)]^2^^31^. Waist circumference (WC) was measured with a flexible measuring tape at a level midway between the lower rib margin^32^. The absolute change in BMI (kg/m^2^) was calculated as BMI_2015_-BMI_2011_ and the relative change in BMI was (BMI_2015_–BMI_2011_)/BMI_2015_. The absolute change in WC (cm) was calculated as WC_2015_-WC_2011_ and the relative change in WC was (WC_2015_–WC_2011_)/WC_2015._ We grouped subjects using two axes of BMI and WC changes and dichotomized them into increase or decrease based on the absolute changes of each axis for analysis. Based on the changes in BMI and WC from the 2011 (Wave1) to the 2015 (Wave3) survey results, they were divided into four groups: both BMI and WC decreased, BMI and WC increased simultaneously, BMI decreased and WC increased, BMI increased and WC decreased. In addition, the data is divided into four equal parts based on changes in BMI and WC: quartile 1 (Q1), quartile 2 (Q2), quartile 3 (Q3), and quartile 4 (Q4), representing 0 to 25%, 26 to 50%, 51 to 75%, and 76 to 100% of BMI and WC indices, respectively, from lowest to highest.

### Covariates

Socio-demographic characteristics including age, sex, education levels, marital status, current residence, smoking, alcohol drinking, taking activities, having regular exercise, and chronic disease were collected by the self-reported questionnaire. These categories have been used extensively in our previous research^33–36^. Age was sorted into 45–54 years, 55–64 years, 65–74 years, and above 75 years old. Education levels were classified into illiterate, less than elementary school, high school, and above vocational school. Marital status was classified into single and married. Current residence included urban and rural. Smoking was categorized into no smoker, former smoker, and current smoker. Alcohol drinking was divided into never drinking, less than once a month, and more than once a month. Taking activities were sorted into ever (at least once a month) and never. Having regular exercise included no exercise, less than exercises, and regular exercises. The counts of chronic disease were classified into 0, 1–2, and 3–14.

### Statistical analysis

Statistical Product Service Solutions (SPSS) software, version 26.0, was used to conduct the analyses (IBM SPSS, Armonk, NY, USA). Socio-demographic characteristics are analyzed by sex and percentages are provided. Chi-square testing was used to compare the distribution of classified variables by sex. The mean and standard deviation were used to express continuous variables. We compared the differences in basic characteristics using the analysis of variance and chi-square test for continuous and categorical parameters, respectively. Under meeting the proportional hazards assumption, Cox regression analysis was used to determine the hazard ratio (HR) and 95% confidence intervals (CIs) of MetS based on the BMI-WC change categories, with the BMI and WC increased simultaneously as the reference. Multivariable adjustments included covariates such as age, sex, educational levels, marital status, current residence, smoking, alcohol drinking, taking activities, having regular exercises, and chronic diseases. Based on the levels of relative and absolute changes in BMI and WC, the relationship between BMI and WC changes and the prevalence of MetS was explored using the quartile method. *P* < 0.05 suggested statistical difference.

## Results

Table 1 shows the baseline characteristics with and without MetS by sex in 2015. A total of 3697 subjects were included in this study, of whom 1988 (53.77%) were male and 1709 (46.23%) were female. According to the results, there are 422 women and 257 men with MetS, respectively. Moreover, we found significant differences in the quartiles of absolute changes in BMI and WC between males and females (*P* < 0.05).

**Table 1:** Baseline characteristics with and without MetS by sex in 2015 (N, %).

Variables	Total (N = 3697,100%)	Male (N = 1988, 53.77%)		*χ2*	*P*	Female (N = 1709, 46.23%)		*χ2*	*P*
		Without MetS	With MetS			Without MetS	With MetS		
N(%)	3697(100.0)	1731(16.76)	257(83.24)			1287(25.44)	422(74.56)		
Age(years)									
45–54	1307(35.4)	474(27.38)	81(31.52)	3.445	0.328	581(45.14)	171(40.52)	10.473	0.015
55–64	1470(39.8)	712(41.13)	105(40.86)			490(38.07)	163(38.63)		
65–74	729(19.7)	436(25.19)	53(20.62)			178(13.83)	62(14.69)		
≥ 75	191(5.2)	109(6.30)	18(7.00)			38(2.95)	26(6.16)		
Education levels									
Illiterate	992(26.8)	245(14.15)	33(12.84)	5.635	0.131	541(42.04)	173(41.00)	0.596	0.897
Less than elementary school	2383(64.5)	1304(75.33)	187(72.76)			666(51.75)	226(53.55)		
High school	230(6.2)	128(7.39)	22(8.56)			62(4.82)	18(4.27)		
Above vocational school	92(2.5)	54(3.12)	15(5.84)			18(1.40)	5(1.18)		
Marital status									
Single	376(10.2)	156(9.01)	21(8.17)	0.195	0.659	141(10.96)	58(13.74)	2.402	0.121
Married	3321(89.8)	1575(90.99)	236(91.83)			1146(89.04)	364(86.26)		
Current residence									
Rural	3500(94.7)	325(95.59)	1523(90.17)	2.407	0.121	1219(94.72)	393(93.13)	1.498	0.221
Urban	197(5.3)	15(4.41)	166(9.83)			68(5.28)	29(6.87)		
Smoking									
No	2058(55.7)	403(23.28)	59(22.96)	4.548	0.103	1202(93.40)	394(93.36)	0.104	0.949
Former smoke	304(8.2)	238(13.75)	48(18.68)			13(1.01)	5(1.18)		
Current smoke	1335(36.1)	1090(62.97)	150(58.37)			72(5.59)	23(5.45)		
Alcohol drinking									
No	2302(62.3)	721(41.65)	103(40.08)	4.093	0.129	1105(85.86)	373(88.39)	2.555	0.279
Less than once a month	315(8.5)	205(11.84)	21(8.17)			73(5.67)	16(3.79)		
More than once a month	1080(29.2)	805(46.50)	133(51.75)			109(8.47)	33(7.82)		
Taking activities									
No	1902(51.4)	874(50.49)	126(49.03)	0.192	0.661	693(53.85)	209(49.53)	2.380	0.123
Yes	1795(48.6)	857(49.51)	131(50.97)			594(46.15)	213(50.47)		
Having regular exercises									
No exercise	2256(61.0)	1069(61.76)	158(61.48)	0.445	0.801	777(60.37)	252(59.72)	0.446	0.800
Less than exercises	769(20.8)	351(20.28)	56(21.79)			275(21.37)	87(20.62)		
Regular exercises	672(18.2)	311(17.97)	43(16.73)			235(18.26)	83(19.67)		
Chronic diseases(counts)									
0	1304(35.3)	610(35.24)	99(38.52)	3.571	0.168	466(36.21)	129(30.57)	4.909	0.086
1–2	1847(50.0)	867(50.09)	113(43.97)			643(49.96)	224(53.08)		
3–14	546(14.8)	254(14.67)	45(17.51)			178(13.83)	69(16.35)		
BMI									
Q1	924(25.0)	458(26.46)	39(15.18)	53.853	0.000	344(26.73)	84(19.91)	21.744	0.000
Q2	924(25.0)	449(25.94)	48(18.68)			333(25.87)	94(22.27)		
Q3	925(25.0)	437(25.25)	60(23.35)			322(25.02)	105(24.88)		
Q4	924(25.0)	387(22.36)	110(42.80)			288(22.38)	139(32.94)		
WC									
Q1	934(25.3)	472(27.27)	24(9.34)	85.707	0.000	367(28.52)	65(15.40)	51.429	0.000
Q2	921(24.9)	441(25.48)	41(15.95)			328(25.49)	92(21.80)		
Q3	935(25.3)	442(25.53)	78(30.35)			322(25.02)	115(27.25)		
Q4	907(24.5)	376(21.72)	114(44.36)			270(20.98)	150(35.55)		

BMI: body mass index; WC: waist circumference.

BMI = weight (kg) / [height (m)]^2^.

The cutoff values of absolute changes in BMI in males: < -0.611 kg/m^2^, -0.611 to < 0.183 kg/m^2^, 0.183 to < 1.007 kg/m^2^, ≥ 1.007 kg/m^2^.

The cutoff values of absolute changes in BMI in females: < -0.511 kg/m^2^, -0.511 to < 0.326 kg/m^2^, 0.326 to < 1.275 kg/m^2^, ≥ 1.275 kg/m^2^.

The cutoff values of absolute changes in BMI in total: < -0.569 kg/m^2^, -0.569 to < 0.251 kg/m^2^, 0.251 to < 1.128 kg/m^2^, ≥ 1.128 kg/m^2^.

The cutoff values of absolute changes in WC in males: < -3.0 cm, -3.0 to < 1.0 cm, 1.0 to < 4.8 cm, ≥ 4.8 cm.

The cutoff values of absolute changes in WC in females: < -2.4 cm, -2.4 to < 1.9 cm, 1.9 to < 5.8 cm, ≥ 5.8 cm.

The cutoff values of absolute changes in WC in total: < -2.7 cm, -2.7 to < 1.3 cm, 1.3 to < 5.3 cm, ≥ 5.3 cm.

Table 2 shows the baseline characteristics according to the changes in BMI and WC. Among the subjects, 907 (24.5%) had decreases in both BMI and WC, 1558 (42.1%) had increases in both BMI and WC, 617 (16.7%) had decreased BMI and increased WC, and 563 (15.2%) had increased BMI and decreased WC.

**Table 2:** Baseline characteristics according to the changes in BMI and WC (N, %).

Variables	Total	BMI↑, WC↑	BMI↑, WC↓	BMI↓, WC↑	BMI↓, WC↓
N (%)	3697(100.0)	1558(42.1)	563(15.2)	617(16.7)	907(24.5)
Sex					
Male	1988(53.8)	811(52.1)	295(52.4)	318(51.5)	540(59.5)
Female	1709(46.2)	747(47.9)	268(47.6)	299(48.5)	367(40.5)
Age(years)					
45–54	1307(35.4)	623(40.0)	181(32.1)	221(35.8)	263(29.0)
55–64	1470(39.8)	599(38.4)	233(41.4)	240(38.9)	382(42.1)
65–74	729(19.7)	280(18.0)	114(20.2)	122(19.8)	199(21.9)
≥ 75	191(5.2)	56(3.6)	35(6.2)	34(5.5)	63(6.9)
Education					
Illiterate	992(26.8)	406(26.1)	159(28.2)	156(25.3)	255(28.1)
Less than elementary school	2383(64.5)	997(64.0)	367(65.2)	407(66.0)	578(63.7)
High school	230(6.2)	111(7.1)	25(4.4)	41(6.6)	52(5.7)
Above vocational school	92(2.5)	44(2.8)	12(2.1)	13(2.1)	22(2.4)
Marital status					
Single	376(10.2)	143(9.2)	59(10.5)	77(12.5)	90(9.9)
Married	3321(89.8)	1415(90.8)	504(89.5)	540(87.5)	817(90.1)
Current residence					
Rural	3500(94.7)	1479(94.9)	541(96.1)	578(93.7)	852(93.9)
Urban	197(5.3)	79(5.1)	22(3.9)	39(6.3)	55(6.1)
Smoking					
No	2058(55.7)	906(58.2)	320(56.8)	343(55.6)	461(50.8)
Former smoke	304(8.2)	111(7.1)	54(9.6)	45(7.3)	88(9.7)
Current smoke	1335(36.1)	541(34.7)	189(33.6)	229(37.1)	358(39.5)
Alcohol drinking					
No	2302(62.3)	973(62.5)	351(62.3)	390(63.2)	550(60.6)
Less than once a month	315(8.5)	133(8.5)	46(8.2)	51(8.3)	82(9.0)
More than once a month	1080(29.2)	452(29.0)	166(29.5)	176(28.5)	275(30.3)
Taking activities					
No	1902(51.4)	811(52.1)	300(53.3)	297(48.1)	467(51.5)
Yes	1795(48.6)	747(47.9)	263(46.7)	320(51.9)	440(48.5)
Having regular exercises					
No exercise	2256(61.0)	984(63.2)	342(60.7)	372(60.3)	521(57.4)
Less than exercises	769(20.8)	321(20.6)	118(21.0)	125(20.3)	195(21.5)
Regular exercises	672(18.2)	253(16.2)	103(18.3)	120(19.4)	191(21.1)
Chronic diseases(counts)					
0	1304(35.3)	584(37.5)	177(31.4)	207(33.5)	318(35.1)
1–2	1847(50.0)	758(48.7)	292(51.9)	331(53.6)	443(48.8)
3–14	546(14.8)	216(13.9)	94(16.7)	79(12.8)	146(16.1)

BMI: body mass index; WC: waist circumference.

Table 3 showed the association between changes in BMI and WC and the risk of MetS. After adjusting for age, sex (total subgroup), educational levels, marital status, current residence, smoking, alcohol drinking, taking activities, having regular exercises, and chronic diseases, the risk of MetS was significantly lower in the group with decreases in both BMI and WC (HR = 0.338; 95% CIs 0.264, 0.433) and the group with increased BMI and decreased WC (HR = 0.375; 95% CIs 0.282, 0.499) when compared with the group with increases in both BMI and WC among the total participants. In addition, the group with decreased BMI and WC had a significantly lower risk of MetS in both males (HR = 0.259; 95% CIs 0.173, 0.388) and females (HR = 0.409; 95% CIs 0.297, 0.563) when compared with the group with increased BMI and WC, respectively.

**Table 3:** Association between changes in BMI and WC and the risk of MetS.

	Total		Male		Female	
	Model 1	Model 2	Model 1	Model 2	Model 1	Model 2
BMI↑, WC↑	Reference	Reference	Reference	Reference	Reference	Reference
BMI↑, WC↓	0.391(0.295,0.518)***	0.375(0.282,0.499)***	0.301(0.185,0.489)***	0.307(0.189,0.501)***	0.446(0.313,0.635)***	0.416(0.291,0.594)***
BMI↓, WC↑	0.674(0.535,0.851)***	0.648(0.511,0.821)***	0.664(0.462,0.954)*	0.683(0.474,0.985)*	0.670(0.493,0.912)*	0.645(0.472,0.881)**
BMI↓, WC↓	0.336(0.264,0.429)***	0.338(0.264,0.433)***	0.252(0.168,0.376)***	0.259(0.173,0.388)***	0.437(0.320,0.599)***	0.409(0.297,0.563) ***

BMI: body mass index; WC: waist circumference; HR: hazard ratio; 95% CIs 95% confidence intervals.

Model 1: unadjusted.

Model 2: adjusted for age, sex (total subgroup), education levels, marital status, current residence, smoking, alcohol drinking, taking activities, having regular exercise, and chronic disease.

^*^P < 0.05, ^**^P < 0.01, ^***^P < 0.001.

Table 4 presents the association between absolute changes in BMI and WC from 2011 to 2015 and the prevalence of MetS in 2015. Compared to the highest quartile, BMI and WC in the lowest quartile were significantly associated with MetS events in both males and females. Among females, after adjusting for all covariates, the lowest quartile BMI (HR = 0.486; 95%CIs 0.354, 0.667) (*P* < 0.05) and WC (HR = 0.303 95%CIs 0.217, 0.424) (*P* > 0.05) were significantly correlated with MetS. Similar results were observed in males, the prevalence of MetS was significant in both BMI (HR = 0.302; 95%CIs 0.204, 0.448) (*P* < 0.05) and WC (HR = 0.170; 95%CIs 0.107, 0.270) (*P* < 0.05).

**Table 4:** Association between the absolute changes of BMI and WC and the prevalence of MetS.

	Absolute changes in BMI		Absolute changes in WC	
	Model 1	Model 2	Model 1	Model 2
Total (N = 3697)				
1st quartile(low)	0.407(0.320,0.519)***	0.411(0.322,0.526)***	0.247(0.190,0.321)***	0.250(0.191,0.326)***
2nd quartile	0.476(0.377,0.601)***	0.488(0.385,0.619)***	0.397(0.314,0.501)***	0.409(0.323,0.518)***
3rd quartile	0.657(0.528,0.818)***	0.677(0.541,0.845)***	0.605(0.488,0.749)***	0.622(0.501,0.774)***
4th quartile(high)	Reference	Reference	Reference	Reference
*p*-trend	1.366(1.265,1.475)***	1.359(1.257,1.469)***	1.590(1.468,1.722)***	1.58(1.457,1.713)***
Male (N = 1988)				
1st quartile(low)	0.300(0.203,0.442)***	0.302(0.204,0.448)***	0.168(0.106,0.266)***	0.170(0.107,0.270) ***
2nd quartile	0.376(0.261,0.542)***	0.381(0.264,0.551)***	0.307(0.209,0.450)***	0.307(0.209,0.450)***
3rd quartile	0.483(0.343,0.681)***	0.474(0.336,0.670)***	0.582(0.423,0.801)***	0.570(0.413,0.786)***
4th quartile(high)	Reference	Reference	Reference	Reference
*p*-trend	1.519(1.342,1.721)***	1.512(1.334,1.715)***	1.810(1.586,2.066)***	1.805(1.579,2.063)***
Female (N = 1709)				
1st quartile(low)	0.506(0.370,0.692) ***	0.486(0.354,0.667) ***	0.319(0.229,0.444)***	0.303(0.217,0.424)***
2nd quartile	0.585(0.431,0.794)***	0.554(0.407,0.755)***	0.505(0.372,0.685)***	0.497(0.365,0.676)***
3rd quartile	0.676(0.501,0.911)*	0.667(0.493,0.902)**	0.643(0.480,0.861)**	0.653(0.486,0.877)**
4th quartile(high)	Reference	Reference	Reference	Reference
*p*-trend	1.252(1.133,1.383)***	1.273(1.151,1.409)***	1.442(1.302,1.598)***	1.466(1.322,1.626)***

BMI: body mass index; WC: waist circumference; HR: hazard ratio; 95% CIs confidence intervals.

Model 1: unadjusted.

Model 2: adjusted for age, sex (total subgroup), education levels, marital status, current residence, smoking, alcohol drinking, taking activities, having regular exercise, and chronic disease.

^*^P < 0.05, ^**^P < 0.01, ^***^P < 0.001.

The cutoff values of absolute changes in BMI in males: < − 0.611 kg/m^2^, − 0.611 to < 0.183 kg/m^2^, 0.183 to < 1.007 kg/m^2^, ≥ 1.007 kg/m^2^.

The cutoff values of absolute changes in BMI in females: < − 0.511 kg/m^2^, − 0.511 to < 0.326 kg/m^2^, 0.326 to < 1.275 kg/m^2^, ≥ 1.275 kg/m^2^.

The cutoff values of absolute changes in BMI in total: < − 0.569 kg/m^2^, − 0.569 to < 0.251 kg/m^2^, 0.251 to < 1.128 kg/m^2^, ≥ 1.128 kg/m^2^.

The cutoff values of absolute changes in WC in males: < − 3.0 cm, − 3.0 to < 1.0 cm, 1.0 to < 4.8 cm, ≥ 4.8 cm.

The cutoff values of absolute changes in WC in females: < − 2.4 cm, − 2.4 to < 1.9 cm, 1.9 to < 5.8 cm, ≥ 5.8 cm.

The cutoff values of absolute changes in WC in total: < − 2.7 cm, − 2.7 to < 1.3 cm, 1.3 to < 5.3 cm, ≥ 5.3 cm.

Table 5 presents the association between relative changes in BMI and WC from 2011 to 2015 and the prevalence of MetS in 2015. Compared to the highest quartile, BMI and WC in the lowest quartile were significantly associated with MetS events in both males and females. Among females, after adjusting for all covariates, the lowest quartile BMI (HR = 0.510; 95%CIs 0.370, 0.702) (*P* < 0.05) and WC (HR = 0.318; 95%CIs 0.225, 0.449) (*P* < 0.05) were significantly correlated with MetS. Similar results were observed in males, the prevalence of MetS was significant in both BMI (HR = 0.307; 95%CIs 0.204, 0.464) (*P* < 0.05) and WC (HR = 0.160; 95%CIs 0.099, 0.259) (*P* < 0.05).

**Table 5:** Association between the relative changes of BMI and WC and the prevalence of MetS.

	Relative changes in BMI		Relative changes in WC	
	Model 1	Model 2	Model 1	Model 2
Total (N = 3697)				
1st quartile(low)	0.411(0.321,0.526)***	0.416(0.323,0.534)***	0.251(0.192,0.329)***	0.257(0.195,0.338)***
2nd quartile	0.549(0.436,0.692)***	0.560(0.443,0.708)***	0.480(0.381,0.604)***	0.503(0.398,0.636)***
3rd quartile	0.747(0.601,0.930)**	0.763(0.611,0.953)*	0.703(0.569,0.870)**	0.745(0.600,0.925)**
4th quartile(high)	Reference	Reference	Reference	Reference
*p*-trend	1.347(1.248,1.454) ***	1.341(1.240,1.449) ***	1.544(1.426,1.672) ***	1.528(1.410,1.656)***
Male (N = 1988)				
1st quartile(low)	0.305(0.203,0.458)***	0.307(0.204,0.464) ***	0.158(0.098,0.255)***	0.160(0.099,0.259)***
2nd quartile	0.491(0.343,0.703)***	0.494(0.345,0.709)***	0.370(0.257,0.533)***	0.370(0.256,0.534)***
3rd quartile	0.647(0.462,0.905)*	0.629(0.448,0.883)**	0.604(0.437,0.834)**	0.590(0.426,0.816)**
4th quartile(high)	Reference	Reference	Reference	Reference
*p*-trend	1.465(1.295,1.657) ***	1.459(1.288,1.653) ***	1.769(1.552,2.015) ***	1.763(1.545,2.012) ***
Female (N = 1709)				
1st quartile(low)	0.531(0.387,0.729) ***	0.510(0.370,0.702)***	0.335(0.238,0.472)***	0.318(0.225,0.449)***
2nd quartile	0.670(0.494,0.909)*	0.641(0.471,0.872)**	0.610(0.450,0.827)**	0.600(0.442,0.816)**
3rd quartile	0.778(0.577,1.049)	0.771(0.570,1.042)	0.838(0.627,1.118)	0.847(0.632,1.135)
4th quartile(high)	Reference	Reference	Reference	Reference
*p*-trend	1.228(1.111,1.356)***	1.248(1.128,1.380)***	1.408(1.271,1.560)***	1.432(1.291,1.588)***

BMI: body mass index; WC: waist circumference; HR: hazard ratio; 95% CIs confidence intervals.

Model 1: unadjusted.

Model 2: adjusted for age, sex (total subgroup), education levels, marital status, current residence, smoking, alcohol drinking, taking activities, having regular exercise, and chronic disease.

^*^P < 0.05, ^**^P < 0.01, ^***^P < 0.001.

The cutoff values of relative changes in BMI in males: < − 0.029 kg/m^2^, − 0.029 to < 0.008 kg/m^2^, 0.008 to < 0.045 kg/m^2^, ≥ 0.045 kg/m^2^.

The cutoff values of relative changes in BMI in females: < − 0.024 kg/m^2^, − 0.024 to < 0.015 kg/m^2^, 0.015 to < 0.055 kg/m^2^, ≥ 0.055 kg/m^2^.

The cutoff values of relative changes in BMI in total: < − 0.027 kg/m^2^, − 0.027 to < 0.011 kg/m^2^, 0.011 to < 0.049 kg/m^2^, ≥ 0.049 kg/m^2^.

The cutoff values of relative changes in WC in males: < − 0.037 cm, − 0.037 to < 0.012 cm, 0.012 to < 0.056 cm, ≥ 0.056 cm.

The cutoff values of relative changes in WC in females: < − 0.031 cm, − 0.031 to < 0.022 cm, 0.022 to < 0.069 cm, ≥ 0.069 cm.

The cutoff values of relative changes in WC in total: < − 0.034 cm, − 0.034 to < 0.016 cm, 0.016 to < 0.063 cm, ≥ 0.063 cm.

## Discussion

We found that among middle-aged and older Chinese individuals, WC and BMI reductions were significantly linked to a lower risk of MetS. It is noteworthy that among subjects with increased BMI and decreased WC (adjusted HR, 0.375), and decreased BMI and WC (adjusted HR, 0.338), the degree of reduction in MetS risk was greater than that of subjects with decreased BMI and increased WC (adjusted HR, 0.648).

Obesity measured through BMI and WC has a higher correlation with inflammation and metabolic markers, which may promote insulin resistance and increase the risk of MetS^37–39^. Moreover, the combination of BMI and WC can provide more information in identifying high-risk individuals with MetS compared to the single index. Epidemiological studies have demonstrated that increasing BMI may not necessarily lead to an increase in MetS risk, as changes in body composition (muscle mass and fat mass) are more vital indicators for assessing MetS risk^40,41^. The difference between muscle mass and fat mass (especially abnormal obesity) may also be significant for the same BMI unit, so single BMI levels may not predict MetS^42^. WC is a human body measurement index that has a strong correlation with visceral fat and subcutaneous fat. At lower BMI levels, WC can also better represent visceral fat^43^. The reduction of WC is beneficial for the improvement of metabolic syndrome via reducing fasting blood glucose, triglycerides, and blood pressure levels, as well as increasing HDL. Even a slight increase in waist circumference can be a risk factor for the development of MetS and its parameters^44^. According to a study investigating the relationship between muscle mass and fat mass and insulin resistance and MetS, it was found that regardless of changes in muscle mass, abdominal fat accumulation is associated with insulin resistance and MetS^45^. The explanation for this phenomenon may be that insulin induced glucose uptake occurs in skeletal muscle, and high muscle mass can stabilize the control of glucose levels, providing a certain protective effect on insulin resistance and MetS^46,47^. Many previous studies^32,41,48^ have also demonstrated a correlation between BMI and WC and MetS.

Although both BMI and WC can be used to evaluate obesity and metabolic disorders, there may be differences in body composition among different races and populations^49^. For example, given a fixed BMI and WC, compared to people with European backgrounds, Chinese people have higher body fat and abdominal fat, as well as higher total cholesterol and cardiovascular metabolic risk factors^50,51^. With an increase in WC, Chinese people may experience more significant changes in metabolic risk factors and face greater cardiovascular risks^52^. Therefore, previous studies^53–55^ have suggested that different BMI or WC thresholds may be necessary to avoid overestimating or underestimating the risk of MetS between different races and populations due to a single BMI and WC threshold.

Due to the lack of scientifically determined critical values for changes in BMI and WC, we investigated the impact of absolute changes in BMI and WC on MetS and found that a decrease in WC and BMI is beneficial for reducing the risk of metabolic syndrome, but there are some gender differences in the degree of reduction in metabolic syndrome risk. Our research results are consistent with some previous studies^42,56,57^. A literature review suggests that females are more likely to suffer from MetS than males due to work pressure and lower socio-economic status^56^. Males under 50 years old had a slightly higher prevalence of MetS, but this difference reverses after 50 years old. Females are more likely to develop MetS as they age, and this tendency may be influenced by their sex as well as other variables such as reduced levels of high-density lipoprotein cholesterol, insulin resistance, postmenopausal abdominal obesity, and hyperandrogenism^9,58^. In addition, we found that an increase in BMI may not be accompanied by an increase in MetS risk, which may be due to the protective effect of higher BMI associated with an increase in muscle mass^42^. Therefore, the sex differences in the risk of MetS may be due to differences in muscle mass content between males and females.

In our cohort study, we also found that changes in BMI and WC levels from 2011 to 2015 were positively correlated with a higher prevalence of MetS. Compared to the highest quartile, we found that after adjusting for all covariates, the quartile with the lowest absolute change in BMI levels had a probability of developing MetS of 0.307 times in males and 0.486 times in females. In addition, we also observed similar results in the absolute changes in WC. After adjusting for covariates, the relationship between relative and absolute changes in BMI, WC, and MetS risk was statistically significant.

Moreover, we found that the reduction in WC may have greater benefits for MetS risk than the reduction in BMI. In the two groups with reduced WC, we found that an increase or decrease in BMI had almost no effect on MetS risk. Whether in males or females, lower WC levels have a stronger protective effect on individuals with MetS. Numerous studies have shown that exercising and changing dietary patterns can reduce visceral and abdominal obesity, and reduce the risk of MetS by reducing WC^44,59,60^.

### Strengths and limitations

The advantage of this study is that the data comes from a nationwide cohort study targeting middle-aged and elderly community residents, with participants aged 45 or above. We compared the impact of changes in BMI and WC on MetS among middle-aged and elderly Chinese. In previous studies, some indicators were used to predict MetS incidence rates, but changes in indicators were not taken into account as an influence on incidence rates. The cohort research design and relatively large sample size further guarantee that causality can be established. Several limitations are present in this study. The first limitation was that many participants were exclusion due to missing data, and more complete data should be collected for future research. Second, we only considered confounding factors that were identified, but there are still unknown factors. Thirdly, the research population is only composed of middle-aged and elderly people in China, and generalizations about other ages and races may be limited. Finally, this study measured and classified exposure during a four-year follow-up period, but the results may be influenced by the length of follow-up time. Although we controlled for confounding factors, there may be a risk of partial bias in the research results, and future prospective cohort studies are needed to validate our findings.

### Ethics approval and consent to participate

All data are openly published as microdata at http://opendata.pku.edu.cn/dataverse/CHARLS with no direct contact with all participants. Approval for this study was given by the medical ethics committee of Wannan Medical College (approval number 2021-3). The patients/participants provided their written informed consent to participate in this study.

## Conclusion

In summary, changes in BMI and WC are significantly correlated with subsequent MetS risk in the middle-aged and elderly population in China. Subjects with reduced BMI and WC benefited the most in reducing the risk of MetS, followed by those with increased BMI and decreased WC. In addition, the reduction in WC may have greater benefits for MetS risk than the reduction in BMI, this may be due to the reduction of the ectopic fat pool and increased skeletal muscle mass.

## Data Availability

Data can be accessed via http://opendata.pku.edu.cn/dataverse/CHARLS.

## Abbreviations

CHARLS: China Health and Retirement Longitudinal Study
MetS: Metabolic syndrome
WC: Waist circumference
BMI: Body mass index
NCEP ATP III: National Cholesterol Education Program-Adult Treatment Panel III
CT: Computer tomography
MRI: Magnetic resonance imaging
TG: Triglycerides
HDL-C: Low high-density lipoprotein cholesterol
SBP: Systolic blood pressure
DBP: Diastolic blood pressure
FPG: Fasting plasma glucose
SPSS: Statistical Product Service Solutions
HR: Hazard ratios
95% CIs: 95% Confidence intervals
SD: Standard deviation
